# A Rare Case of Low-Grade Appendiceal Mucinous Neoplasm Presenting As Mucinous Vaginal Discharge

**DOI:** 10.7759/cureus.73858

**Published:** 2024-11-17

**Authors:** Evan M Eggiman, Chris Henson, Desmond Ponder, Nancy Collins

**Affiliations:** 1 Osteopathic Medicine, Campbell University School of Osteopathic Medicine, Lillington, USA; 2 Family Medicine, Conway Medical Center, Myrtle Beach, USA; 3 Obstetrics and Gynecology, Conway Medical Center, Myrtle Beach, USA

**Keywords:** appendiceal mucinous neoplasm, mucinous discharge, pelvic pain, pseudomyxoma peritonei, right-sided hemicolectomy

## Abstract

Appendiceal mucinous neoplasms (AMNs) are rare tumors that often present with non-specific symptoms, posing diagnostic challenges. This report aims to emphasize the importance of considering AMNs in the differential diagnosis of atypical pelvic symptoms, especially when initial evaluations suggest gynecological issues. A 56-year-old female with a past medical history of hyperlipidemia and a total vaginal hysterectomy performed over 20 years prior for abnormal uterine bleeding due to fibroids presented to her gynecologist with a three-week history of vaginal discharge and pelvic pain. Initial gynecological assessments were unremarkable, prompting a CT scan of the abdomen and pelvis, which revealed a markedly dilated, fluid-filled appendix, indicative of a mucinous neoplasm. The patient underwent laparoscopic right hemicolectomy, which confirmed a low-grade AMN with clear margins. Post-surgical follow-up showed no recurrence, and a surveillance colonoscopy one year later revealed a benign tubular adenoma. This case highlights the need to consider AMNs in the differential diagnosis of pelvic symptoms, particularly when initial gynecological evaluations do not identify a clear cause. AMNs can present with symptoms that overlap with those of gynecological conditions, making accurate diagnosis challenging. Effective management through early recognition and surgical intervention is important to prevent complications. This report demonstrates the value of comprehensive diagnostic evaluation and the importance of including rare tumors in the differential diagnosis of atypical pelvic symptoms.

## Introduction

Appendiceal mucinous neoplasms (AMNs) are rare tumors and account for less than 1% of gastrointestinal cancers [1]. AMNs can be further classified into low-grade AMN (LAMN) and high-grade AMN (HAMN), distinguished primarily by the degree of epithelial dysplasia [2]. AMNs typically feature villous or flat neoplastic mucinous epithelium, often associated with lymphoid tissue atrophy, resembling adenomas [3]. The clinical course is usually indolent initially and may be discovered incidentally, but it can become more aggressive in later stages [4]. Surgical treatment, including appendectomy or hemicolectomy, is the mainstay of management, depending on the extent of the neoplasm [1,5]. The purpose of this study is to report a case in which a mucinous vaginal discharge led to the diagnosis, treatment, and subsequent surveillance of LAMN.

## Case presentation

A 56-year-old female with a past medical history of hyperlipidemia and a total vaginal hysterectomy performed over 20 years ago for abnormal uterine bleeding due to fibroids presented to her gynecologist with a three-week history of intermittent vaginal discharge and pelvic pain. She had undergone an unremarkable annual gynecological exam a few weeks prior, including a pelvic exam and bimanual exam. The patient reported a brownish mucoid discharge that was noticeable upon wiping but not substantial enough to stain her undergarment liner. She denied any history of vaginal trauma, dyspareunia, or changes in bowel or bladder habits. Given her nonspecific symptoms, along with her age, history of hysterectomy, recent benign gynecological exam, and the sudden onset of symptoms, a CT of the abdomen and pelvis was ordered.

**Figure 1:**
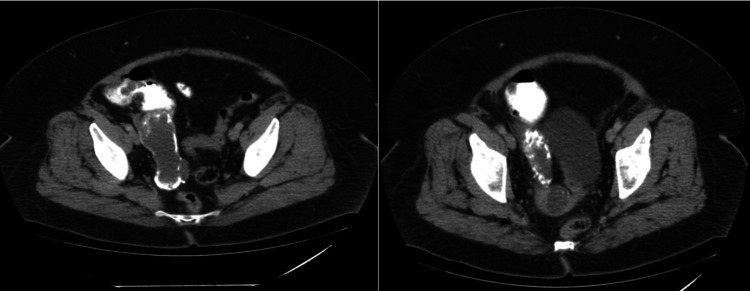
CT scan showing a markedly dilated, predominantly fluid-filled appendix consistent with a mucinous neoplasm.

A CT scan of the abdomen and pelvis with contrast was performed, which revealed a markedly dilated, predominantly fluid-filled appendix measuring 12.2 × 4.6 × 5.0 cm, most consistent with a mucinous neoplasm (Figure 1). The scan also showed a small intermediate-density lesion or fluid collection at the cranial aspect of the vagina/cervix. The patient was referred to surgery for further evaluation and management.

After considering the imaging findings and the size of the lesion, a laparoscopic right hemicolectomy was deemed the most appropriate surgical approach. This decision was based on the need to ensure complete resection of the tumor while preserving as much healthy tissue as possible.

During the procedure, appropriate anatomical landmarks were identified. The tumor-involved right colon and a portion of the terminal ileum were resected, and the healthy small and large bowel segments were anastomosed. Intraoperative exploration of the abdomen did not reveal any palpable evidence of metastatic disease or other abnormalities. The entire specimen was submitted to pathology for evaluation.

**Figure 2:**
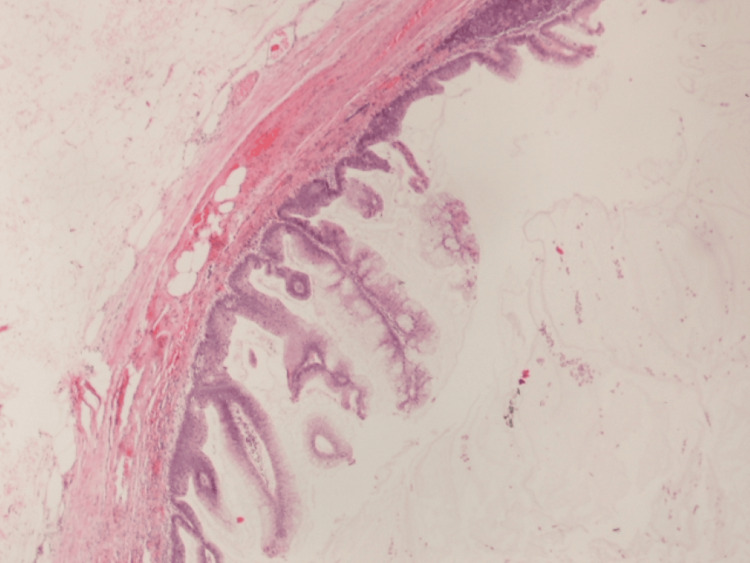
Histopathological image of low-grade appendiceal mucinous neoplasm This hematoxylin and eosin-stained section shows mucin-filled cystic dilation of the appendiceal lumen, fibrotic changes in the appendiceal wall, and mucin dissecting through the muscularis propria.

**Figure 3:**
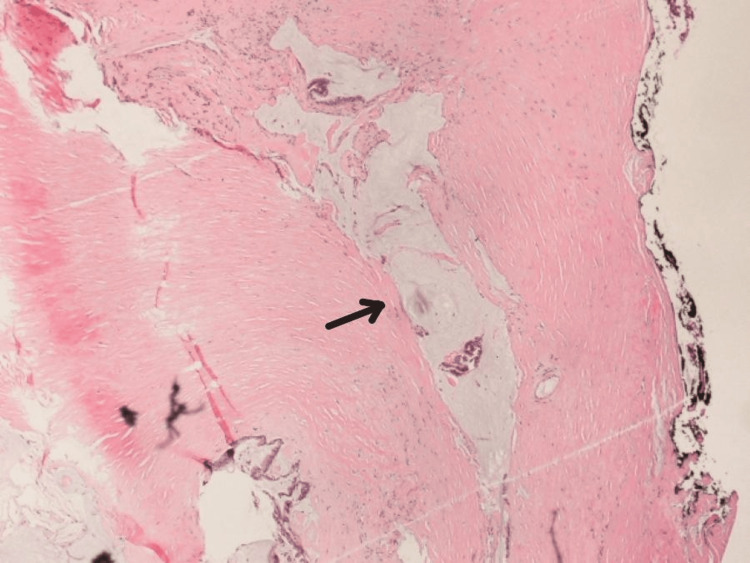
Histopathological image of low-grade appendiceal mucinous neoplasm with pushing invasion This hematoxylin and eosin-stained section demonstrates a low-grade appendiceal mucinous neoplasm disrupting the appendiceal wall through a pushing invasion (arrow). A pushing invasion is characterized by a smooth, broad front as the tumor expands into nearby tissues rather than by aggressive, infiltrative growth. The mucinous epithelium does not show signs of infiltrative growth, showing clear margins.

The pathology report confirmed a LAMN with clear margins and no evidence of malignancy. The tumor extended through the lamina propria and subserosa but did not involve the serosal surface (Figure 2 and Figure 3). Pathologic staging was pT3pN0 with benign colon and small bowel. At the follow-up appointment with the surgical team, the patient had no complaints and was doing well. Oncology evaluation recommended continued observation. A surveillance colonoscopy performed one year post-procedure revealed a benign tubular adenoma.

## Discussion

Appendiceal mucinous lesions, both benign and neoplastic, exhibit a slight female predominance and are typically diagnosed in patients in their 50s and 60s [1,3]. The clinical course of LAMN is generally indolent early on but can become more aggressive in later stages. Clinical presentations of LAMN can vary widely, ranging from asymptomatic cases to those with severe symptoms. It is estimated that 15-20% of LAMNs are discovered incidentally due to their asymptomatic nature [4].

Other patients may exhibit symptoms resembling appendicitis, including right lower abdominal pain, fever, nausea, and vomiting [6]. If mucin accumulates in the appendix, a mass may be palpable, or the condition might be incidentally discovered during appendectomies or abdominal imaging performed for other reasons [1].

In advanced stages, patients may experience intermittent cramping pain, intestinal obstruction due to the mass, ovarian metastasis, and urinary symptoms from ureteral compression [1,7]. Some more severe clinical features include rupture and spread of mucin and neoplastic epithelium into the peritoneum, causing mucinous ascites and pseudomyxoma peritonei [1,5]. Given the potential severity of these complications, it is essential to consider a broad differential diagnosis in postmenopausal women presenting with mucinous vaginal discharge, as this may be an indicator of malignancy.

The mucinous vaginal discharge observed in this case may be explained by mucin leakage from the LAMN into the peritoneal cavity. Although no fistulous tract was directly identified on the CT scan, mucin from the appendix had extended into the peritoneal cavity and localized around the superior aspect of the vaginal cuff. The patient’s history of a total vaginal hysterectomy may have created an area of minor resistance or scarring, allowing for mucin accumulation in this location. From there, the mucin could intermittently drain through the vaginal cuff, leading to the observed discharge. While most LAMNs are asymptomatic and found incidentally, sudden growth or rupture of the lesion may explain her recent onset of mucinous discharge and pelvic pain symptoms [4,8]. The presentation of mucinous vaginal discharge in conjunction with LAMN is rare, with the exact prevalence unknown. Only a few cases have been reported in the literature [8-10].

In this case, the patient’s nonspecific symptoms of vaginal discharge and pelvic pain initially suggested a gynecological origin. This is consistent with a similar case where LAMN presenting as mucoid vaginal discharge was initially misdiagnosed as a myxoid uterine tumor [9]. In extremely rare instances, pseudomyxoma peritonei resulting from an initial LAMN can spread and involve the endometrium, leading to pelvic pain and mucinous vaginal discharge [9,10]. In such cases, the most definitive diagnosis is achieved through surgical removal and histopathological examination [9,10]. Given the patient’s history of a hysterectomy, imaging was performed, which incidentally uncovered the underlying LAMN.

The differential diagnosis for a postmenopausal woman with mucinous vaginal discharge should include endometrial or cervical cancer, ovarian tumors, and infectious etiologies [11,12]. However, when common gynecological causes are excluded, LAMN should be considered, particularly when imaging reveals an appendiceal mass. This case underscores the importance of considering gastrointestinal sources in the differential diagnosis of atypical gynecological symptoms, particularly in postmenopausal women.

## Conclusions

AMNs are rare but potentially serious tumors that can present with asymptomatic or vague symptoms, such as pelvic pain, vaginal discharge, or signs mimicking appendicitis. Given the nonspecific nature of these symptoms, AMNs should be considered in the differential diagnosis for women in their 50s and 60s who present with unexplained pelvic symptoms or mucinous ascites. While initial imaging, such as CT scans, can provide valuable insights, definitive diagnosis requires histopathological examination through biopsy or surgical resection. The treatment approach is determined by the tumor’s grade and extent, with LAMNs typically managed by complete surgical resection of the appendix and involved bowel segments. In cases of HAMNs with peritoneal spread, more aggressive interventions, including cytoreductive surgery and hyperthermic intraperitoneal chemotherapy, may be necessary. Early diagnosis and appropriate management are important to prevent complications and improve patient outcomes.
